# UV and visible light photocatalytic activity of Au/TiO_2_ nanoforests with Anatase/Rutile phase junctions and controlled Au locations

**DOI:** 10.1038/srep41253

**Published:** 2017-01-24

**Authors:** Yang Yu, Wei Wen, Xin-Yue Qian, Jia-Bin Liu, Jin-Ming Wu

**Affiliations:** 1 State Key Laboratory of Silicon Materials and School of Materials Science and Engineering, Zhejiang University, Hangzhou 310027, P. R. China; 2 College of Mechanical and Electrical Engineering, Hainan University, Haikou, 570228, P. R. China

## Abstract

To magnify anatase/rutile phase junction effects through appropriate Au decorations, a facile solution-based approach was developed to synthesize Au/TiO_2_ nanoforests with controlled Au locations. The nanoforests cons^®^isted of anatase nanowires surrounded by radially grown rutile branches, on which Au nanoparticles were deposited with preferred locations controlled by simply altering the order of the fabrication step. The Au-decoration increased the photocatalytic activity under the illumination of either UV or visible light, because of the beneficial effects of either electron trapping or localized surface plasmon resonance (LSPR). Gold nanoparticles located preferably at the interface of anatase/rutile led to a further enhanced photocatalytic activity. The appropriate distributions of Au nanoparticles magnify the beneficial effects arising from the anatase/rutile phase junctions when illuminated by UV light. Under the visible light illumination, the LSPR effect followed by the consecutive electron transfer explains the enhanced photocatalysis. This study provides a facile route to control locations of gold nanoparticles in one-dimensional nanostructured arrays of multiple-phases semiconductors for achieving a further increased photocatalytic activity.

Semiconductor photocatalysis utilizes natural sun light or artificial light sources to initiate catalytically specific redox reactions under mild conditions, which finds wide applications in environmental remediation, photocatalytic water-splitting, and CO_2_ reduction^1^. The emergence of novel photocatalysts^2^,^3^,^4^,^5^ in recent years does not hinder researchers’ enthusiasm on titanium dioxide (TiO_2_), which is one of the most traditional yet promising semiconductors for photocatalytic applications because of its exceptional merits of biological friendliness, chemical inertness, low cost, and earth-abundance^1^. The high charge recombination rate and the wide band gap (3.0–3.2 eV) impose a fundamental restriction on the overall photocatalytic efficiency for TiO_2_^1^, which are the focus of numerous studies ever since Fujishima and Honda’s pioneer work on TiO_2_ photocatalysis^6^.

Decorating TiO_2_ with noble metal nanoparticles such as Pt^7^, Ag^8^, Pd^9^, and Au^10^,^11^,^12^,^13^ is an effective tactic to improve the photocatalytic activity. The noble metal nanoparticles contact closely with TiO_2_ to form Schottky barriers, which drive photogenerated electrons from the n-type TiO_2_ to the noble metals and enhance the charge separation rate and the photocatalytic activity. For TiO_2_ decorated with Au and Ag, there is an additional effect, the localized surface plasmon resonance (LSPR), which contributes to a strong absorption of the visible light and thus the photocatalytic performance under the visible light illumination^13^,^14^,^15^. The LSPR effect is affected readily by shape^16^, size^17^,^18^,^19^, and content^20^ of Au nanoparticles, as well as characteristics of the TiO_2_ supports^13^,^18^,^21^,^22^.

Energy band engineering is another interesting topic for improving photocatalytic activity of TiO_2_^23^. The anatase/rutile phase junction has been argued to favor the charge separation of TiO_2_^24^,^25^,^26^. Compositing TiO_2_ with other semiconductors possessing either a wider or a narrower band gap also results in efficient charge separations and/or enhanced light harvesting from the solar light^27^,^28^,^29^. Not surprisingly, such TiO_2_-based composite semiconductors or TiO_2_ with mixed phases could be decorated with noble metals to further enhance the photocatalytic performance^13^,^14^,^22^. Recently, the importance of the architecture of Au/TiO_2_ nanoparticles has been noted under the visible light illumination^13^,^14^.

When compared with nanoparticles, one-dimensional (1D) semiconducting nanostructures such as nanorods and nanowires have been found to enhance the photocatalytic activity of TiO_2_ with distinct charge transport capabilities^30^,^31^. Imposing branches on the surface of well-aligned 1D nanostructures to form branched nanowire arrays, also termed as nanotrees or nanoforests, further increases the performance because of the enhanced active sites and light harvesting capability^31^,^32^,^33^,^34^. In practical photocatalysis for wastewater treatments, TiO_2_ thin films avoid the nuisance powder recovering procedure. It is thus of great importance to develop TiO_2_ thin films with high photocatalytic activity. Based on the literature, it can be anticipated that decorating noble metals on controlled locations in TiO_2_ nanoforests could achieve enhanced photoelectrochemical performances.

In this work, an architectural design was implemented to study the possible effects of the location of Au nanoparticles decorated on TiO_2_ nanoforests with anatase/rutile phase junctions. Simply altering the order of the fabrication step (Fig. 1), Au nanoparticles were located preferably either on the interface of anatase/rutile phase junctions, or on rutile branches, which in turn affected readily the photocatalytic efficiency towards photodegradations of rhodamine B in water under the illumination of either UV or visible light. Efforts were made to clarify the possible mechanisms that cause the distinct photocatalytic activity.

## Results and Discussions

### Morphology and phase characterizations

**Figure 1:**
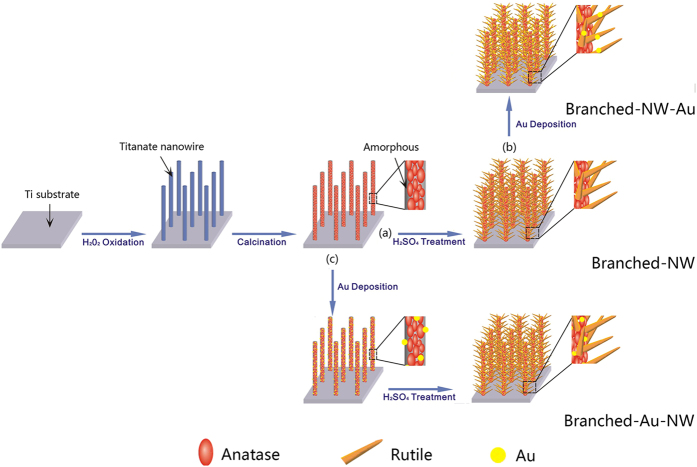
A schematic diagram showing the formation of the branched TiO_2_ nanowires (**a**, Branched-NW), those after the Au-loading (**b**, Branched-NW-Au), and the branched TiO_2_ nanowires with an intermediate Au-loading (**c**, Branched-Au-NW).

**Figure 2:**
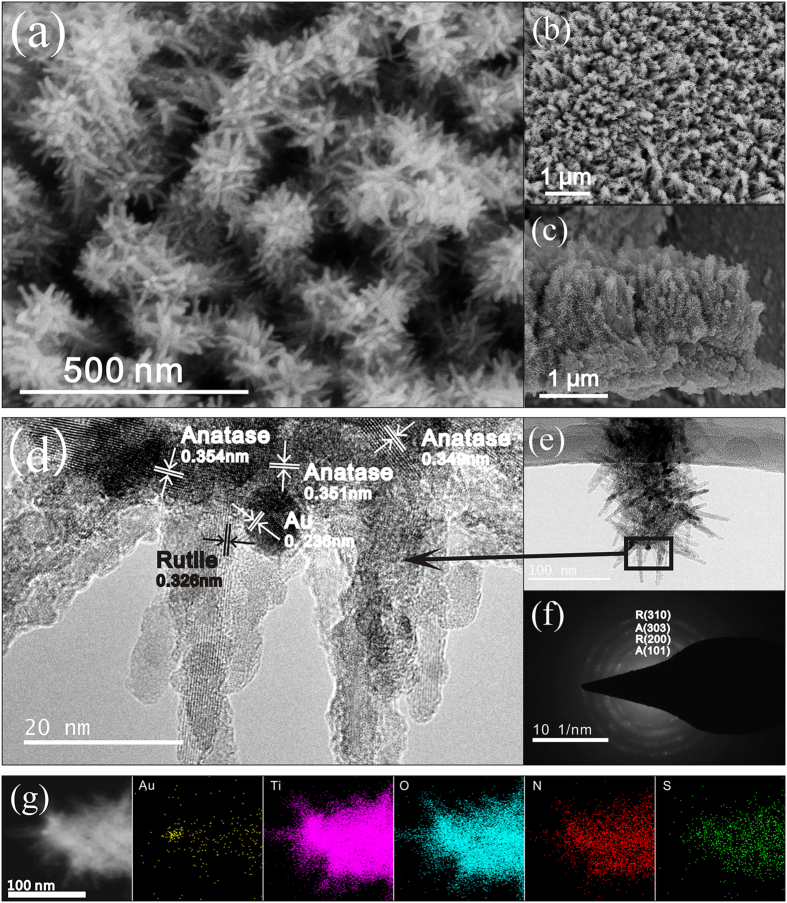
(**a**) High and (**b**) low magnification top view, (**c**) cross-sectional FESEM images of the Branched-Au-NW; (**d**) HRTEM, (**e**) low-magnification TEM, and (**f**) the corresponding SAED of the Branched-Au-NW; (**g**) TEM image of another Branched-Au-NW and the corresponding EDS mapping images of Au, Ti, O, N and S.

Supplementary Figure S1 shows FESEM images of the TiO_2_ nanoforests and those after Au-loading at various locations. It can be seen that quasi-aligned 1D branched nanowires covered homogeneously the Ti substrates. The Au-loading procedures induced no significant change in morphologies. Figure 2 illustrates FESEM, TEM, HRTEM, selected area electron diffraction (SAED), and EDS analysis results of the Branched-Au-NW (refer to Fig. 1 for the sample ID). The thickness of the TiO_2_ nanoforests is ca. 1 μm. An intermediate layer ca. 1 μm in thickness, which consisted of compact nanoparticles, can also be seen between the top layer and the substrate (Fig. 2a–c).

The low-magnification TEM image shows clearly a typical branched nanowire that is decorated with Au nanoparticles (Fig. 2e). The average diameter of the nanorod branch is ca. 10 nm and the length is ca. 45 nm; while the Au particles have an approximately spherical shape that is 8–9 nm in diameter. The HRTEM image (Fig. 2d) exhibited that the backbone is formed by many tiny crystal grains with a lattice spacing of ca. 0.35 nm, which can be attributed to the (101) facet of anatase TiO_2_. The fringe with inter-plane spaces of ca. 0.32 nm on the branch can be discerned, which is attributed to the (110) crystal plane of rutile TiO_2_. Locating between the anatase backbone and the rutile branch, fringes with inter-plane spaces of ca. 0.235 nm can be seen clearly, which can be assigned to the (111) facet of Au. Hence, the HRTEM observations suggest that, for the Branched-Au-NW specimen, the well-crystalized single-crystalline rutile nanorods grew along the polycrystalline anatase TiO_2_ nanowires with an arbitrary angular orientation; whilst most Au nanoparticles located at the interface between the backbone and the branch, which contacted closely with both anatase and rutile. Figure 2f demonstrates the SAED pattern of the branched nanowire shown in Fig. 2e. Multi-rings characteristic of a mixture of anatase and rutile can be discerned, which is in good accordance with the HRTEM observations (Fig. 2d). The EDS mapping (Fig. 2g) suggests the homogenous distributions of Ti, O, N, and S throughout the Branched-Au-NW. A spot corresponding to an Au nanoparticle located between the anatase trunk and rutile branch can be discerned.

**Figure 3:**
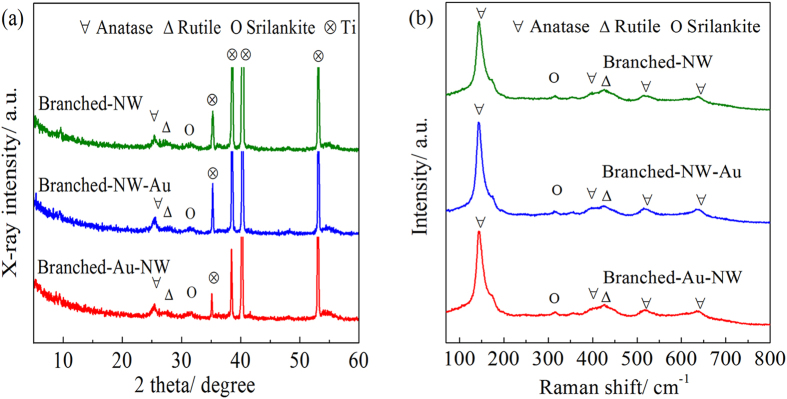
(**a**) XRD patterns and (**b**) Raman spectra of the Branched-NW, Branched-NW-Au, and Branched-Au-NW.

The crystal structure of the TiO_2_ nanoforests can find support from both XRD patterns and Raman spectra. XRD peaks corresponding to both anatase and rutile can be discerned, besides those arising from the metallic Ti substrates (Fig. 3a). It is not surprising that three specimens exhibited similar XRD patterns, indicating that the phase structure of TiO_2_ does not change after the deposition of gold nanoparticles. Trace srilankite TiO_2_ can also be discerned. The corresponding Raman spectrum suggests more clearly the coexistence of anatase, rutile, and srilankite (Fig. 3b). Both XRD (Fig. 3a) and SAED patterns (Fig. 2f) exhibit no signals corresponding to Au, which can be contributed to the minor amounts of Au nanoparticles. It is noted that only one peak appeared in both XRD patterns and Raman spectra, which is a weak proof of the existence of srilankite TiO_2_. However, a previous study^35^ revealed that, the srilankite TiO_2_ was detected on a low temperature derived TiO_2_ thin film. In that case, Raman peaks were identified at 168, 314, 354 and 425 cm^−1^, which have been contributed to srilankite TiO_2_. Also, the XRD peak located at ca. 31.5° was identified. Therefore, we believe it is safe to ascribe the present XRD peak located at ca. 31.5° (Fig. 3a) and the Raman peak located at ca. 314 cm^−1^ (Fig. 3b) to srilankite TiO_2_.

### Au contents

The atomic ratio of Au/Ti was measured by ICP-MS to be 0.36% and 0.51%, for the specimens of Branched-Au-NW and Branched-NW-Au, respectively. By using the defined Au-loading parameters (identical HAuCl_4_ solution and photo- reduction time), the Branched-Au-NW contained less Au nanoparticles when compared with the Branched-NW-Au. This may be contributed to the different phase compositions of the TiO_2_ films before Au-decorations. For the Branched-Au-NW, Au-loading was performed on poorly crystallized anatase TiO_2_ nanowires; whilst for the Branched-NW-Au, Au-loading was conducted on TiO_2_ nanoforests consisted of rutile TiO_2_ branches that grow radially around the poorly crystallized anatase TiO_2_ trunk^34^. It is well established that the anatase/rutile phase junctions facilitate charge separations^24^,^25^,^26^ and hence the photocatalytic efficiency for the Au-loading, which explained the higher content of gold nanoparticles decorated on Branched-NW-Au. It is also argued that Au nanoparticles form more easily on the rutile surface because a number of oxygen vacancies act as the crystal nucleation sites^13^.

**Figure 4:**
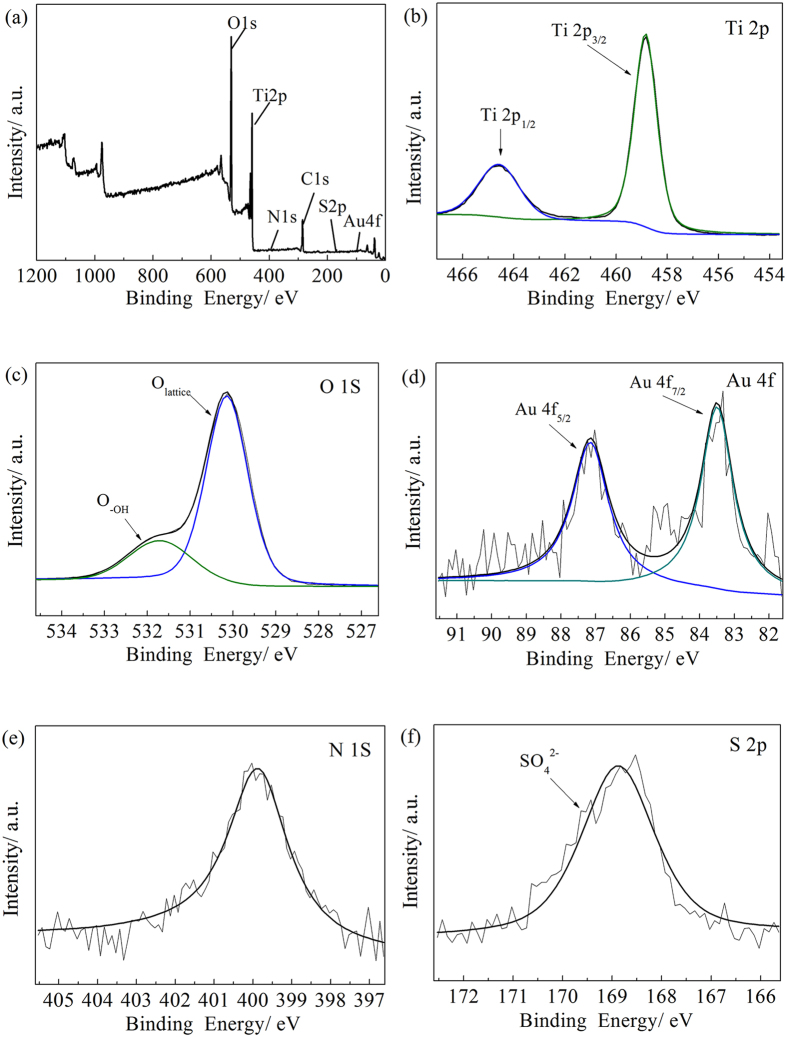
(**a**) Wide scan and high-resolution XPS spectra of (**b**) Ti 2p, (**c**) O 1 s, (**d**) Au 4 f, (**e**) N 1 s, and (**f**) S 2p for the Branched-Au-NW.

The surface compositions of the Branched-Au-NW were analyzed by XPS. Figure 4a shows that the surface layer of the Branched-Au-NW is composed of Ti, O, Au, N, and S, which is consistent to the EDS mapping images (Fig. 2g). The XPS spectrum of Ti 2p exhibits two dominant peaks, which correspond to Ti 2p_1/2_ at 464.6 eV and Ti 2p_3/2_ at 458.8 eV, indicating that Ti exist in the form of Ti^4+^. The separation between the two peaks is 5.8 eV (Fig. 4b), which is in agreement with the XPS data in the literature^7^. As shown in Fig. 4c, the O 1 s spectrum can be fitted by two components: a higher binding energy (BE) peak near the 531.7 eV, originating from the hydroxyl group (-OH)^36^, and a lower BE, in the vicinity of 530.1 eV, originating from the crystal lattice oxygen (Ti–O–Ti).

The XPS spectrum of Au can be separated to two peaks, with a lower BE at 83.4 eV and a high BE at 87.1 eV, corresponding to Au 4f_7/2_ and Au 4f_5/2_, respectively (Fig. 4d). The typical Au 4f_7/2_ peak locates at 84.0 eV^37^ for bulk metallic gold; the slight shift in BE to a lower value can be ascribed to the redistribution of the electrons at the Au-TiO_2_ contact interfaces, because of the difference in the work function between Au (5.27 eV) and TiO_2_ (4.1 eV). This is an indication that Au nanoparticles interact with the adjacent TiO_2_^14^,^37^,^38^. The electron transferring from TiO_2_ to Au nanoparticles is therefore facilitated, which increases the valence charge density of Au atoms and reduces the binding energy of Au in the TiO_2_ film.

The binding energy of N 1 s locates at ca. 399.8 eV, which can be assigned to nitrogen species bonding to various surface oxygen sites N-O, or N-N, and N-C bonds (Fig. 4e)^39^. The incorporation of N into the Branched-Au-NW film is believed to result from the decomposition of melamine during the fabrication of the titanate nanowires. After the H_2_SO_4_ treatment, sulfate ions also incorporate into the TiO_2_ film, which gave the XPS peak at 168.9 eV (Fig. 4f)^36^.

Table 1 lists the surface compositions of the two Au-decorated TiO_2_ nanoforests derived by the XPS analysis. The atomic Au/Ti ratio determined by the XPS analysis is ca. 0.27% for Branched-Au-NW, which is lower than that of Branched-NW-Au. The atomic Au/Ti ratios evaluated by XPS roughly agreed with that derived by the ICP-MS measurement.

### Growth of the TiO_2_ Nanoforests with Controlled Au-location

**Figure 5:**
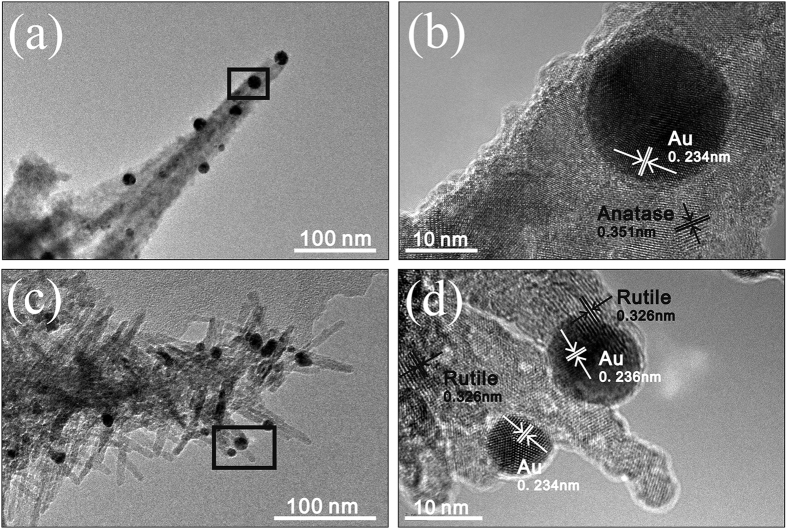
(**a**,**b**) TEM and HRTEM images of the Au-decorated nanowires just before the final H_2_SO_4_ treatment; (**c**,**d**) TEM and HRTEM images of the Branched-NW-Au.

Figure 5a,b shows that, Au nanoparticles were intimately decorated on the surface of the poorly crystallized anatase TiO_2_ nanowires before the final H_2_SO_4_ treatment. That is to say, in the current tactic to synthesize Branched-Au-NW, Au nanoparticles were firstly deposited on the surface of the nanowires which were just subjected to the intermediate calcination. During the final H_2_SO_4_ treatment, the poorly crystallized anatase TiO_2_ nanowires were partly attacked by H_2_SO_4_, which released hydrated Ti(IV) ions into the acid solution^34^. Once the Ti(IV) ions accumulated to a critical concentration, nucleation and subsequent growth of rutile TiO_2_ branches around the anatase TiO_2_ trunk occurred. It seems that the edges formed between the nanowires and the decorated Au nanoparticles provide heterogeneous nucleation sites for the TiO_2_ branches, which resulted in the preferred locations of the Au nanoparticles around the junctions of the anatase trunk and rutile branch.

When the Au-decoration procedure was moved to be the final step, the Au nanoparticles did not locate preferably on the anatase/rutile phase junctions any more. Because of certain “tip” effects, most of Au nanoparticles located on the surface of the rutile branch (Fig. 5c,d), rather than on the interface of rutile branch and anatase backbone. In addition, for the Branched-NW film, because the anatase trunk is surrounded by rutile branches, Au nanoparticles had a higher possibility to sit on rutile. Therefore, simply altering the Au-loading order fulfilled the control in the location of Au nanoparticles, which affects readily the resultant photocatalytic performance of the Au-decorated TiO_2_ nanoforests, as will be discussed later.

### PL and UV-Vis DRS Characterizations

**Figure 6:**
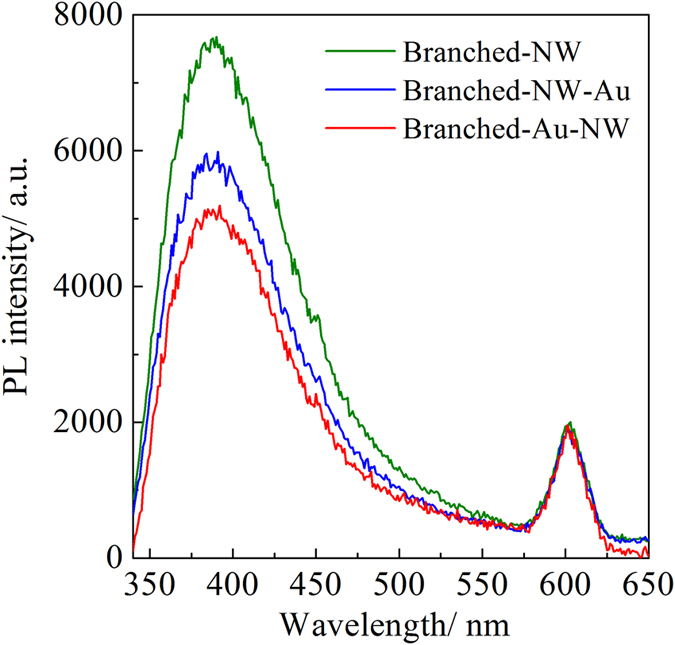
Ambient PL spectra of the Branched-NW, Branched-NW-Au, and Branched-Au-NW.

Figure 6 shows the PL spectra of the three specimens, all of which possessed a feature that consists of two emission peaks in the UV-visible range. The UV emission centered at 400 nm is related to the electron transition from the valence band and the conduction band^38^, and the emission centered at around 608 nm may arise from the recombination of photo-generated holes with the electrons in singly occupied oxygen vacancies^40^. It can be seen that, the intensity of UV emission decreased in the order of Branched-NW, Branched-NW-Au, and Branched-Au-NW. Therefore, the Au-decoration suppresses the charge recombination, which is closely related to the location of Au nanoparticles.

**Figure 7:**
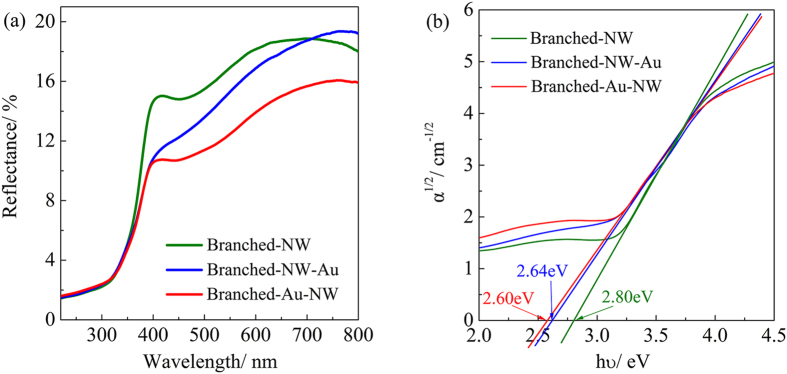
(**a**) UV-Vis diffuse reflectance spectra of the Branched-NW, Branched-NW-Au, and Branched-Au-NW; (**b**) the spectra in an *α*^1/2^~*hν* coordinate to evaluate the band gap.

Figure 7a illustrates the UV-Vis diffuse reflectance spectra collected from the three specimens. The Branched-Au-NW film exhibited the lowest reflectance over the visible light region, which suggests the highest visible light harvesting capability when compared with the Branched-NW and the Branched-NW-Au. The increasing absorption can be ascribed to the LSPR effect arising from Au nanoparticles. A strong oscillation of the metal’s surface free electrons with the varying electric field of the incident light absorbs the energy of photon and conveys to electrons to form surface plasmons, which decay to hot electron-hole pairs; as a result, the light response of the Au-decorated specimens in the visible light region is enhanced^41^,^42^.

Assuming an indirect transition between band gaps, the band gaps of TiO_2_ can be estimated by extrapolating the tangent line in the plot of α^1/2^ against hυ^43^, where α is the absorption coefficient and the hυ is the photon energy. Figure 7b demonstrates that the Branched-Au-NW possessed an indirect band gap of 2.61 eV, which is lower than the value of 2.64 eV and 2.80 eV determined for the Branched-NW-Au and Branched-NW, respectively. The relatively lower band gap of 2.80 eV for Branched-NW, when compared with bulk TiO_2_ (3.2 eV for anatase and 3.0 eV for rutile), can be contributed to the N-doping (Fig. 4e) and the significant oxygen deficiency (Fig. 4c) arising from the low-temperature synthesis route. A DFT calculation by Jia *et al*. revealed that, the band gap of a N, S-codoped TiO_2_ can be narrowed to be. 2.77 eV^44^. The further red-shift for the Au-decorated films was attributed mainly to the interaction of Au and TiO_2_, which might introduce an intra-gap level inside the band gap of TiO_2_^37^. Considering the relatively lower Au content for the Branched-Au-NW (0.36%) when compared with the Branched-NW-Au (0.51%), as determined by ICP-MS, both the higher light harvesting capability and the slightly lower band gap once again convinced the importance of the Au-location. A rough explanation for the enhanced LSPR arising from Au nanoparticles in Branched-Au-NW could be that the contacting surface area between Au and TiO_2_ is higher than that in Branched-NW-Au.

### Photocatalytic Activity

Photocatalytic activities of the three TiO_2_ nanoforests were evaluated by decomposing rhodamine B in water under UV and visible light illuminations, respectively. In absence of any photocatalysts, about 95% and 97% dye molecules remained after 60 min of UV and visible light illuminations. The dark absorption capacity is enhanced after the Au-decoration, which can be ascribed to the interaction of Au and dye molecules. For comparison purpose, thin films of Degussa P25 TiO_2_ nanoparticles (ca. 3.0 μm in thickness, refer to the literature^45^ for the fabrication route), which are generally adopted as a benchmark, were also subjected to the photocatalytic activity evaluations under the identical conditions.

**Figure 8:**
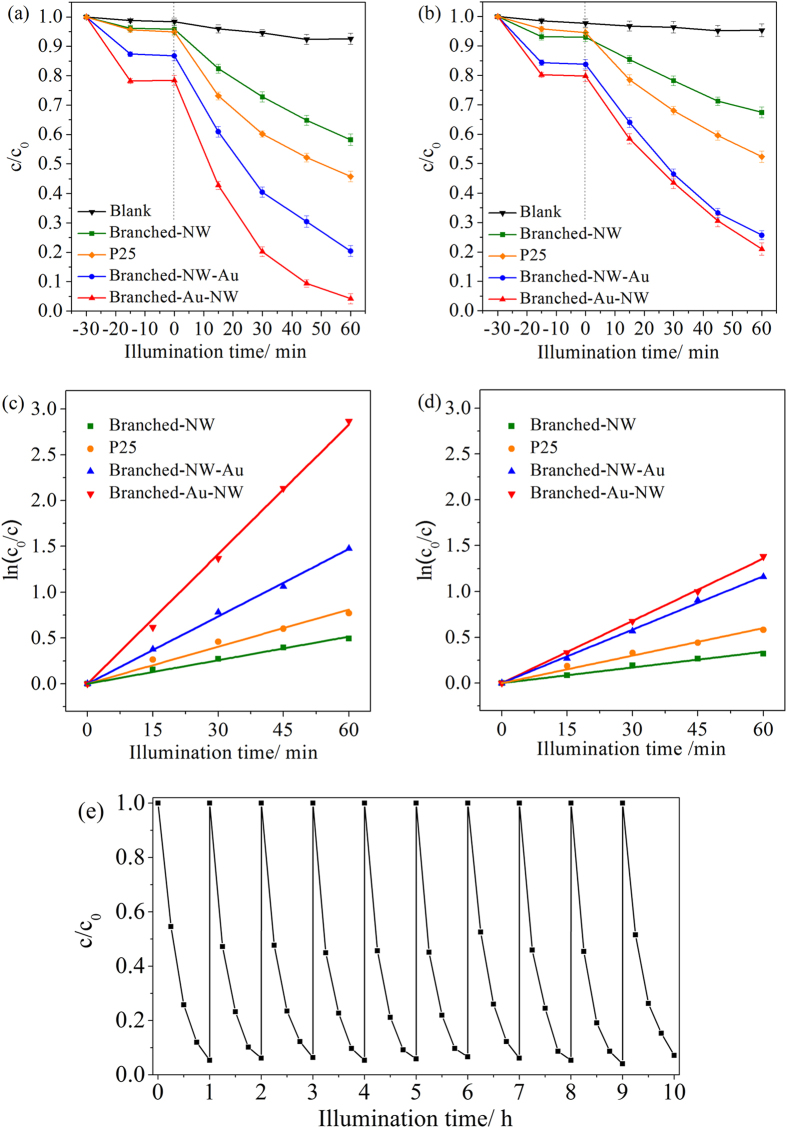
Photodegradation curves of rhodamine B in water in the presence of the P25, Branched-NW, Branched-NW-Au, and Branched-Au-NW, under (**a**) UV light, and (**b**) visible light illumination. (**c**,**d**) Represent the corresponding fitting results assuming a pseudo-first order reaction. The cycling performance of the Branched-Au-NW is illustrated in (**e**), under the UV light illumination.

The HAuCl_4_ concentrations adopted for the Au-decoration were firstly optimized (Supplementary Figure S2). It illustrates that, for both Au-decorated films, there is an optimum HAuCl_4_ concentration (0.040 mM). It can thus be inferred that, certain amounts of Au nanoparticles introduced to the TiO_2_ nanoforests enhanced the photocatalytic activity. However, excess Au nanoparticles aggregate to serve as recombination centers for photogenerated charges, leading to an inferior performance^46^. Figure 8a,b indicates the photodegradation curves, which can be fitted well assuming a pseudo-first-order kinetics^47^,


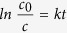


where *c* is the dye concentration after illumination for a duration *t, c*_*0*_ is the dye concentration after the dark adsorption, and *k* is the pseudo-first-order reaction rate constant, which can be obtained by the slope of the straight lines through zero. Figure 8c,d shows the corresponding fitting results and Table 2 lists the reaction rate constants, which were derived using the average data obtained from three repetitive tests. Under the UV light illumination, the reaction rate constant increased from 0.86 to 2.5 × 10^−2^ min^−1^ after the Au-decoration of the Branched-NW. Simply controlling the location of the Au nanoparticles to distribute mainly along the anatase/rutile phase junctions, the reaction rate constant further increased to 4.7 × 10^−2^ min^−1^, which is nearly 5 times higher when compared with the Branched-NW specimen. The Au location affects also the photocatalytic activity under the visible light illumination.

**Figure 9:**
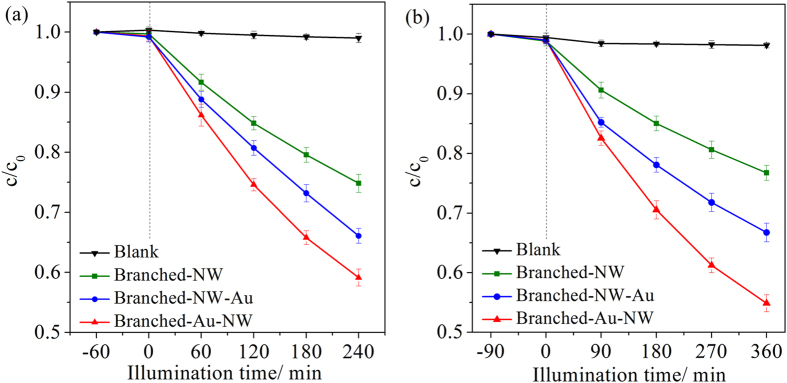
Photodegradation curves of (**a**) *p*-nitrophenol and (**b**) phenol under UV light illumination.

Photocatalytic degradations of *p*-nitrophenol and phenol under the UV light illumination, in the presence of the various Au/TiO_2_ films were also evaluated. Figure 9 and Table 2 show that, the same trend can be discerned. Therefore, it can be concluded that, the photocatalytic activity of the branched TiO_2_ film was enhanced after the Au decoration and the position of Au nanoparticles really has a great impact on the photocatalytic activity. Due to the insufficient efficiency of the present Au/TiO_2_ films under the visible light illumination, the photocatalytic degradations of *p*-nitrophenol and phenol under visible light is not presented in the current investigation. Photosensitive materials are argued to be not suitable as probe chemicals for photocatalytic activity tests, especially those for evaluation of activity under visible light^48^. Further study is thus demanded to disclose the effects of Au-locations on photodegradations of organics besides rhodamine B molecules.

The trapping experiment was employed to disclose the possible photocatalysis mechanism (Supplementary Figure S3). It is inferred that OH•, h^+^, and O_2_•^−^ all contribute to the photocatalytic reaction. The superoxide radicals O_2_•^−^ are the major active species responsible for this photocatalytic oxidation reaction, followed by the photogenerated holes h^+^, and the hydroxyl radical OH•.

### Mechanism

**Figure 10:**
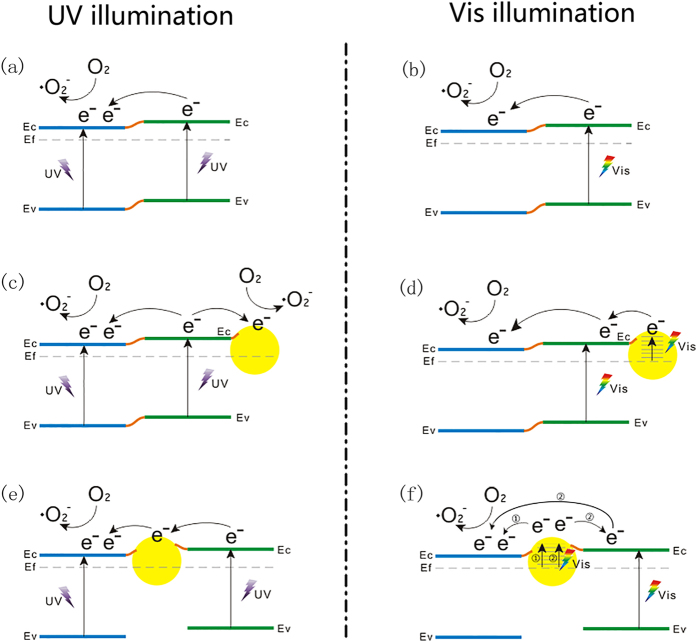
Schematic images showing the enhanced photocatalytic efficiency arising from the intermediate Au-loading for the TiO_2_ nanoforests under the illumination of (**a**,**c**,**e**) UV and (**b**,**d**,**f**) visible light: (**a**,**b**) Branched-NW; (**c**,**d**) Branched-NW-Au; (**e**,**f**) Branched-Au-NW. For simplicity, the degradation route *via* photogenerated holes h^+^ and hydroxyl radicals OH• is not shown.

Figure 10 provides a possible explanation for the beneficial effects arising from Au nanoparticles located between the anatase/rutile phase junctions in the present 1D branched TiO_2_ nanowires. Because Supplementary Figure S3 indicates that superoxide radicals O_2_•^−^ contribute the most to the photocatalytic reaction; only the degradation route via O_2_•^−^ is presented in Fig. 10. It is well established that anatase/rutile phase junction effectively suppresses the recombination of photogenerated electron-hole pairs^30^,^49^,^50^,^51^; however, the exact charge transfer direction still remains a controversy^25^,^52^. Herein, the energetic alignment of the band edges of the anatase and rutile polymorphs of TiO_2_ suggested recently by Scanlon *et al*. is adopted^52^.

### UV Light Illumination

As illustrated in Fig. 10a, under UV light irradiation, photogenerated electrons transfer from rutile to anatase, which results in an enhanced charge separation rate and hence improved photocatalytic activity. In addition, it has been proved that O_2_ reduction by the photo-induced electrons on the rutile surface is inefficient because of the low affinity between the surface and O_2_. In contrast, anatase is more active for O_2_ reduction^13^. As a result, the electron transfer from rutile to anatase accelerates the photodegradation procedure, especially for which superoxide radicals O_2_•^−^ play the key role (Supplementary Figure S3).

In case that the TiO_2_ nanoforests were subjected to a final Au-decoration (Branched-NW-Au), Au nanoparticles prefer to locate on rutile surfaces (Fig. 5c,d). Because of the Shottky barrier between Au and TiO_2_ semiconductor, the photoexcited electrons from rutile will also transfer to Au nanoparticles^13^,^14^, except for the migration to anatase. Thus, Au nanoparticles act as the electrons accepting species at the Au/rutile interface, suppressing further the recombination of photogenerated charges (Fig. 10c). The reaction rate constant thus increased from 0.86 to 2.5 × 10^−2^ min^−1^ (UV + RhB, Table 2). When Au nanoparticles are loaded on the interface between anatase and rutile in the TiO_2_ nanoforests, as for the Branched-Au-NW, they provide a path for rapid and effective electron migrations from rutile to anatase^53^, which significantly enhances the positive effects arising from the anatase/rutile phase junction (Fig. 10e). Such a function is more effective for the charge separation, contributing to an even higher reaction rate constant of 4.7 × 10^−2^ min^−1^ (UV + RhB, Table 2). The enhanced charge separation is supported by the PL measurement (Fig. 6).

### Visible Light Illumination

In the current investigation, the Branched-NW possessed a band gap of 2.80 eV, which corresponds to a wavelength of ca. 440 nm (Fig. 7); therefore, under the illumination of visible light (>420 nm), it is possible for the rutile branch to absorb the photons with appropriate energy to initiate the photocatalytic reaction (Fig. 10b). The additional electrons arising from the LSPR effect of Au nanoparticles, which may also transfer to anatase to initiate the photodegradation reaction there (Fig. 10d), explains the increased reaction rate constant from 0.57 × 10^−2^ min^−1^ for Branched-NW to 1.9 × 10^−2^ min^−1^ for Branched-NW-Au (Vis + RhB, Table 2). The further enhanced reaction rate constant for Branched-Au-NW (2.3 × 10^−2^ min^−1^) is in good accordance with Tsukamoto *et al*.^13^. Here, Au nanoparticles locate preferably at the interface of anatase/rutile, upon the visible light illumination, the LSPR induced electrons transfer from Au nanoparticles to the tightly bound rutile and then through the phase junction to well-conjugated anatase, where the electrons react with surface adsorbed O_2_ to form O_2_•^−^ to assist photodegradations of rhodamine B in water (Path 2, Fig. 10f)^13^,^14^. Because Au nanoparticles contact with both anatase and rutile (Fig. 2d), we believe that, photogenerated electrons arising from the LSPR effect may also transfer directly to the adjacent anatase, which additionally contribute to the enhanced photocatalytic activity (Path 1, Fig. 10f).

### Cycling Performance and mineralization capability

Long-term stability is a major concern of photocatalysts. Figure 8e shows the cycling performance of the Branched-Au-NW film. For up to 10 cycles, no remarkable decay can be discerned, which evidenced the excellent stability of the present Au-decorated TiO_2_ nanoforests. The present Branched-Au-NW film is also capable of inducing deep mineralization of organics in water after UV illumination for certain durations. A total organic carbon (TOC) reduction of ca. 64.2% was achieved for the rhodamine B solution after the UV light illumination for 1 h when assisted by the Branched-Au-NW film. The liquid chromatography (LC) spectra (Supplementary Figure S4), which were utilized to determine the phenol’s concentration during the photodegradation procedure under the UV illumination, shows that, although the P25 film has a higher efficiency on photodegradations of phenol, less by-products can be discerned for the Au/TiO_2_ photocatalyst (Branched-Au-NW). This further supports the capability of the present Au/TiO_2_ photocatalyst to achieve a deep mineralization of organics in water.

## Conclusion

Titania nanoforests, which consisted of anatase nanowires surrounded by radially grown rutile branches, were synthesized on metallic Ti substrates through multi-steps of H_2_O_2_ oxidation, intermediate calcination, and sulfuric acid treatment. A photo-reduction technique was then applied to decorate Au nanoparticles. Altering the order in the fabrication step is effective in controlling the preferred location of Au nanoparticles. Under the UV light illumination, Au nanoparticles, which located preferably at the interface of anatase/rutile, magnify the beneficial effects arising from the anatase/rutile phase junctions; whilst under the visible light illumination, the LSPR effect followed by the consecutive electron transfer results in improved photocatalysis. This finding evidenced the feasibility to further improve photocatalytic activity of multiple-phases semiconductor arrays with one-dimensional nanostructures through carefully controlling the location of noble metals.

## Methods

### Synthesis of TiO_2_ Nanoforests

Figure 1 demonstrates schematically the three steps in sequence to synthesize TiO_2_ nanoforests (abbreviated as **Branched-NW** hereafter) on metallic Ti substrates, that is, H_2_O_2_ oxidation, intermediate calcination, and H_2_SO_4_ treatment^34^. In a typical synthesis, each cleaned Ti plate (5 × 5 × 0.01 cm^3^ in size) was immersed in 50 mL of 8.8 M H_2_O_2_ solution containing 16 mM melamine (C_3_H_6_N_6_) and 29 mM HNO_3_, which was maintained at 80 °C for 48 h to grow hydrogen titanate (H_2_Ti_5_O_11_) nanowire array on the surface. The Ti plate was then taken out, rinsed in sequence with ethanol and deionized water, and subjected to an intermediate thermal treatment in air at 260 °C for 1 h to decompose H_2_Ti_5_O_11_ to amorphous TiO_2_ embedded with poorly crystallized anatase. The Branched-NW was achieved by a final treatment in 50 mL of 5 mM H_2_SO_4_ aqueous solution at 80 °C for 48 h, which induced the formations of rutile TiO_2_ branches that grow radially around the poorly crystallized anatase TiO_2_ trunk^34^.

### Decoration of Au Nanoparticles at Controlled Locations

Au nanoparticles were decorated on Branched-NW via a photo-reduction method^54^. 0.44 mg of HAuCl_4_ (corresponding to an initial concentration of ca. 0.040 mM), methanol (0.2 mL), and deionized water (34 mL) were mixed in a beaker. Branched-NW was immersed into the solution, which was bubbled with pure nitrogen gas, and then subject to the UV light (5.0 mW/cm^2^) irradiation for 5 min. The specimens were dried at 80 °C in an oven overnight, which were designated as **Branched-NW-Au** hereafter. To vary the location of the Au nanoparticles, in a parallel experiment, the order in Au-deposition and H_2_SO_4_ treatment was exchanged. The specimens were noted as **Branched-Au-NW**.

### Characterizations

Surface morphology of thin films was observed using a field emission scanning electron microscopy (FESEM, Hitachi S-4800) and a transmission electron microscopy (TEM, FEI, Tecnai G2 F20 S-TWIN, with EDS capabilities). To prepare the samples for TEM characterizations, the TiO_2_ nanoforests were detached off from the Ti plate and then placed on a carbon pre-coated copper grid. The X-ray diffraction (XRD) tests were conducted on a Rigaku D/max-3B diffractmeter with Cu Kα radiation (λ = 0.154056 nm), operated at 40 kV, 36 mA. Raman spectra were collected using an Almega dispersive Raman system (Nicolet) and a Nd:YAG intracavity doubled laser operating at 532 nm with an incident power of 10 mW. The atomic ratio of Au and Ti was measured with inductively coupled plasma mass spectrometry (ICP-MS, XSeries II, Thermo Fisher Scientific, USA). For the ICP-MS measurement, the Au-decorated TiO_2_ nanoforests were scratched off and dissolved in aqua regia. The X-ray photoelectron spectroscopy (XPS) spectra were collected using an ESCA spectrometer (S-Probe ESCA SSX-100S, Fisons Instrument) and monochromatized Al Kα X-ray (1486.8 eV) irradiation. The binding energy was normalized to the C 1 s energy (284.6 eV) for adventitious hydrocarbons as the indirect standard. The UV-Vis diffuse reflectance spectra were measured using a UV-Vis near-infrared spectrometer (UV-3150, Shimadzu). The ambient photoluminescence (PL) emission spectra characterizations were carried out on a fluorescence spectrophotometer (HITACHI F-4500) with an excited wavelength of 360 nm.

### Photocatalytic Activity Evaluations

Titania thin films (2.5 × 2.5 cm^2^ in size) were used to assist photocatalytic degradations of rhodamine B, *p*-nitrophenol and phenol in water (25 mL) in a Pyrex reactor with a water jacket, which is illustrated schematically in Supplementary Figure S5. Three repetitive tests were conducted to give the average value and error bar (standard deviation) as illustrated in the corresponding degradation curves. The initial concentration of rhodamine B is 0.005 mmol/L, while the initial concentrations of *p*-nitrophenol and phenol are 5 ppm and 10 ppm, respectively. The UV irradiation was provided by an 18 W UV lamp and the visible light by a 500 W Xe-lamp with a 420 nm UV-cut filter. The UV and visible light intensity reaching the sample was measured to be ca. 5.0 mW/cm^2^ and 200.0 mW/cm^2^, respectively, using irradiance meters (model: UV-A and FZ-A, Beijing Normal university, China, measured for the wavelength of 365 nm for UV light and 400–1000 nm for the visible light). The solution was stirred and exposed to air during the photocatalytic reaction. The change in the rhodamine B and *p*-nitrophenol concentrations was monitored with a UV-Vis spectrophotometer (UV-1800PC, Shanghai Mapada, Shanghai, China) at a wavelength of 553 nm and 317 nm, respectively. The phenol concentration was analyzed by liquid chromatography on a Wufeng LC100 (WondaCract C18-ODS column, and 50% CH_3_OH aqueous solution as an eluent). The total organic carbon (TOC) of rhodamine B was measured with a Total Organic Carbon Analyzer (Multi N/C 3100).

To investigate the effects of the active species generated during the photocatalytic reaction under the UV illumination and in the presence of the Branched-Au-NW, the free radicals capture experiments were conducted^55^. The three major oxidants involved in photodegradations of organics in water, that is, hydroxyl radical (OH•), hole (h^+^), and superoxide radical (O_2_•^−^), were trapped by adding 0.5 mL of tert-butanol (t-BuOH), 0.1 mM of ammonium oxalate (AO), and 0.5 mM of 1, 4-benzoquinone (BQ), respectively, into the rhodamine B solutions.

## Additional Information

**How to cite this article:** Yu, Y. *et al*. UV and visible light photocatalytic activity of Au/TiO_2_ nanoforests with Anatase/Rutile phase junctions and controlled Au locations. *Sci. Rep.* **7**, 41253; doi: 10.1038/srep41253 (2017).

**Publisher's note:** Springer Nature remains neutral with regard to jurisdictional claims in published maps and institutional affiliations.

## Supplementary Material

Supplementary Information

## Figures and Tables

**Table 1:** Surface compositions (in at. %) of Au-decorated TiO_2_ nanoforests obtained by XPS.

	C	N	S	O	Au	Ti	Au/Ti by XPS	Au/Ti by ICP
Branched-NW-Au	13.71	0.88	0.56	56.30	0.14	28.41	0.49%	0.51%
Branched-Au-NW	25.71	0.97	1.10	49.70	0.06	22.46	0.27%	0.36%

**Table 2:** Reaction rate constants (*k*, × 10^−2^ min^−1^) for the different organics in the presence of various TiO_2_ nanoforests and under the illumination of UV and visible light.

Films	P25	Branched-NW	Branched-NW-Au	Branched-Au-NW
UV + RhB	1.4	0.86	2.5	4.7
UV+ *p*-nitrophenol	/	0.11	0.16	0.22
UV+ phenol	/	0.07	0.11	0.17
Vis + RhB	1.0	0.57	1.9	2.3

## References

[b1] Chen X. & Mao S. S. Titanium Dioxide Nanomaterials: Synthesis, Properties, Modifications, and Applications. Chem. Rev. 107, 2891–2959 (2007). 17590053 10.1021/cr0500535

[b2] Moniz S. J. A., Shevlin S. A. & Martin D. J. Visible-Light Driven Heterojunction Photocatalysts for Water Splitting–A Critical Review. Energ. Environ. Sci. 8, 731–759 (2015).

[b3] Xiang Q., Cheng B. & Yu J. Graphene-Based Photocatalysts for Solar-Fuel Generation. Angew. Chem. Int. Ed. 54, 11350–11366 (2015). 10.1002/anie.201411096 26079429

[b4] Ghosh S., Kouamé N. A. & Ramos L. Conducting Polymer Nanostructures for Photocatalysis under Visible Light. Nat. mater. 14, 505–511 (2015). 25774954 10.1038/nmat4220

[b5] Bridewell V. L., Alam R. & Karwacki C. J. CdSe/CdS Nanorod Photocatalysts: Tuning the Interfacial Charge Transfer Process through Shell Length. Chem. Mater. 27, 5064–5071 (2015).

[b6] Fujishima A. & Honda K. Electrochemical Photolysis of Water at A Semiconductor Electrode. Nature 238, 37–38 (1972). 12635268 10.1038/238037a0

[b7] Banerjee B., Amoli V. & Maurya A. Green Synthesis of Pt-Doped TiO_2_ Nanocrystals with Exposed (001) Facets and Mesoscopic Void Space for Photo-Splitting of Water under Solar Irradiation. Nanoscale 7, 10504–10512 (2015). 26008203 10.1039/c5nr02097b

[b8] Choi Y., Kim H. & Moon G. Boosting up the Low Catalytic Activity of Silver for H_2_ Production on Ag/TiO_2_ Photocatalyst: Thiocyanate as A Selective Modifier. ACS Catal. 6, 821–828 (2016).

[b9] Fujiwara K., Müller U. & Pratsinis S. E. Pd Subnano-Clusters on TiO_2_ for Solar-Light Removal of NO. ACS Catal. 3, 1887–1893 (2016)

[b10] Zhang D. Q. . Au Nanoparticles Enhanced Rutile TiO_2_ Nanorod Bundles with High Visible-Light Photocatalytic Performance for NO Oxidation. Appl. Catal. B-Environ. 147, 610–616 (2014).

[b11] Nalbandian M. J. . Tailored Synthesis of Photoactive TiO_2_ Nanofibers and Au/TiO_2_ Nanofiber Composites: Structure and Reactivity Optimization for Water Treatment Applications. Environ. Sci. Technol. 49, 1654–1663 (2015). 25582552 10.1021/es502963t

[b12] McEntee M., Stevanovic A., Tang W. J., Neurock M. & Yates J. T. Electric Field Changes on Au Nanoparticles on Semiconductor Supports-the Molecular Voltmeter and Other Methods to Observe Adsorbate-Induced Charge-Transfer Effects in Au/TiO_2_ Nanocatalysts. J. Am. Chem. Soc. 137, 1972–1982 (2015). 25610956 10.1021/ja511982n

[b13] Tsukamoto D. . Gold Nanoparticles Located at the Interface of Anatase/Rutile TiO_2_ Particles as Active Plasmonic Photocatalysts for Aerobic Oxidation. J. Am. Chem. Soc. 134, 6309–6315 (2012). 22440019 10.1021/ja2120647

[b14] Wen Y., Liu B. T., Zeng W. & Wang Y. H. Plasmonic Photocatalysis Properties of Au Nanoparticles Precipitated Anatase/Rutile Mixed TiO_2_ Nanotubes. Nanoscale 5, 9739–9746 (2013). 23963545 10.1039/c3nr03024e

[b15] Cheng H., Fuku K. & Kuwahara Y. Harnessing Single-Active Plasmonic Nanostructures for Enhanced Photocatalysis under Visible Light. J. Mater. Chem. A 3, 5244–5258 (2015).

[b16] Ma L. . Synthesis of Dumbbell-Like Gold-Metal Sulfide Core-Shell Nanorods with Largely Enhanced Transverse Plasmon Resonance in Visible Region and Efficiently Improved Photocatalytic Activity. Adv. Funct. Mater. 25, 898–904 (2015).

[b17] Zielinska-Jurek A. . Preparation and Characterization of Monometallic (Au) and Bimetallic (Ag/Au) Modified-Titania Photocatalysts Activated by Visible Light. Appl. Catal. B-Environ. 101, 504–514 (2011).

[b18] Kimura K., Naya S. & Jin-nouchi Y. TiO_2_ Crystal Form-Dependence of the Au/TiO_2_ Plasmon Photocatalyst’s Activity. J. Phys. Chem. C 116, 7111–7117 (2012).

[b19] Murdoch M., Waterhouse G. I. N. & Nadeem M. A. The Effect of Gold Loading and Particle Size on Photocatalytic Hydrogen Production from Ethanol over Au/TiO_2_ Nanoparticles. Nat. Chem. 3, 489–492 (2011). 21602866 10.1038/nchem.1048

[b20] Tanaka A., Sakaguchi S. & Hashimoto K. Preparation of Au/TiO_2_ with Metal Cocatalysts Exhibiting Strong Surface Plasmon Resonance Effective for Photoinduced Hydrogen Formation under Irradiation of Visible Light. ACS Catal. 3, 79–85 (2012).

[b21] Bian Z., Tachikawa T. & Zhang P. Au/TiO_2_ Superstructure-Based Plasmonic Photocatalysts Exhibiting Efficient Charge Separation and Unprecedented Activity. J. Am. Chem. Soc. 136, 458–465 (2013). 24308587 10.1021/ja410994f

[b22] Priebe J. B., Radnik J. & Lennox A. J J. Solar Hydrogen Production by Plasmonic Au–TiO_2_ Catalysts: Impact of Synthesis Protocol and TiO_2_ Phase on Charge Transfer Efficiency and H_2_ Evolution Rates. ACS Catal. 5, 2137–2148 (2015).

[b23] Li H., Zhou Y. & Tu W. State-of-the-Art Progress in Diverse Heterostructured Photocatalysts toward Promoting Photocatalytic Performance. Adv. Funct. Mater. 25, 998–1013 (2015).

[b24] Kafizas A., Wang X. & Pendlebury S. R. Where Do Photo-Generated Holes Go in Anatase: Rutile TiO_2_? A Transient Absorption Spectroscopy Study of Charge Transfer and Lifetime. J. Phys.Chem. A 120, 715–723 (2016). 26777898 10.1021/acs.jpca.5b11567

[b25] Zhao W. N., Zhu S. C. & Li Y. F. Three-Phase Junction for Modulating Electron–Hole Migration in Anatase–Rutile Photocatalysts. Chem. Sci. 6, 3483–3494 (2015). 10.1039/c5sc00621j PMC5659171 29511511

[b26] Sun X., Dai W. & Wu G. Evidence of Rutile-to-Anatase Photo-Induced Electron Transfer in Mixed-Phase TiO_2_ by Solid-State NMR Spectroscopy. Chem. Commun. 51, 13779–13782 (2015). 10.1039/c5cc04971g 26235480

[b27] Zhang Y., Pei Q. & Liang J. Mesoporous TiO_2_-Based Photoanode Sensitized by BiOI and Investigation of Its Photovoltaic Behavior. Langmuir 31, 10279–10284 (2015). 26327463 10.1021/acs.langmuir.5b02248

[b28] Han C., Wang Y. & Lei Y. *In Situ* Synthesis of Graphitic-C_3_N_4_ Nanosheet Hybridized N-Doped TiO_2_ Nanofibers for Efficient Photocatalytic H_2_ Production and Degradation. Nano Res. 8, 1199–1209 (2015).

[b29] Liu L., Yang W. & Sun W. Creation of Cu_2_O@ TiO_2_ Composite Photocatalysts with p–n Heterojunctions Formed on Exposed Cu_2_O Facets, Their Energy Band Alignment Study, and Their Enhanced Photocatalytic Activity under Illumination with Visible Light. ACS Appl. Mater. Inter. 7, 1465–1476 (2015). 10.1021/am505861c 25546838

[b30] Ge. M. . A Review of One-Dimensional TiO_2_ Nanostructured Materials for Environmental and Energy Applications. J. Mater. Chem. A 4, 6772–6801 (2016).

[b31] Sheng X., He D., Yang J., Zhu K. & Feng X. Oriented Assembled TiO_2_ Hierarchical Nanowire Arrays with Fast Electron Transport Properties. Nano Lett. 14, 1848–1852 (2014). 24628675 10.1021/nl4046262

[b32] Wu W. Q., Xu Y. F., Rao H. S., Su C. Y. & Kuang D. B. Multistack Integration of Three-Dimensional Hyperbranched Anatase Titania Architectures for High-Efficiency Dye-Sensitized Solar Cells. J. Am. Chem. Soc. 136, 6437–6445 (2014). 24725076 10.1021/ja5015635

[b33] Liu C., Tang J., Chen H. M., Liu B. & Yang P. A Fully Integrated Nanosystem of Semiconductor Nanowires for Direct Solar Water Splitting. Nano Lett. 13, 2989–2992 (2013). 23647159 10.1021/nl401615t

[b34] Wu J. M. & Yin J. X. A Facile Solution-Based Approach to A Photocatalytic Active Branched One-Dimensional TiO_2_ Array. RSC Adv. 5, 3465–3469 (2015).

[b35] Sun J. & Wu J. M. A Comparative Study on Photocatalytic Activity of Titania Nanowires Subjected to High-Temperature Calcination and Low-Temperature HCl Treatment. Sc. Adv. Mater. 5, 549–556 (2013).

[b36] Sun J., Wen W. & Wu J. M. Low-Temperature Transformation of Titania Thin Films from Amorphous Nanowires to Crystallized Nanoflowers for Heterogeneous Photocatalysis. J. Am. Ceram. Soc. 96, 2109–2116 (2013).

[b37] Zhu Y. F. . Fabrication and Photoelectrochemical Properties of ZnS/Au/TiO_2_ Nanotube Array Films. Phys. Chem. Chem. Phys. 15, 4041–4048 (2013). 23400011 10.1039/c3cp43572e

[b38] Su F. L. . Dendritic Au/TiO_2_ Nanorod Arrays for Visible-Light Driven Photoelectrochemical Water Splitting. Nanoscale 5, 9001–9009 (2013). 23864159 10.1039/c3nr02766j

[b39] Li D. Z. . New Synthesis of Excellent Visible-Light TiO_2−*x*_N_*x*_ Photocatalyst Using a Very Simple Method. J. Solid State Chem. 180, 2630–2634 (2007).

[b40] Lai L. L. & Wu J. M. A Facile Solution Approach to W, N Co-Doped TiO_2_ Nanobelt Thin Films with High Photocatalytic Activity. J. Mater. Chem. A 3, 15863–15868 (2015).

[b41] Zhang X. M., Chen Y. L., Liu R. S. & Tsai D. P. Plasmonic Photocatalysis. Rep. Prog. Phys. 76, 4 (2013). 10.1088/0034-4885/76/4/046401 23455654

[b42] Zhang X., Liu Y. & Kang Z. H. 3D Branched ZnO Nanowire Arrays Decorated with Plasmonic Au Nanoparticles for High-Performance Photoelectrochemical Water Splitting. ACS Appl. Mat. Inter. 6, 4480–4489 (2014). 10.1021/am500234v 24598779

[b43] Sanchez E. & Lopez T. Effect of the Preparation Method on the Band Gap of Titania and Platinum-Titania Sol-Gel Materials. Mater. Lett. 25, 271–275 (1995).

[b44] Jia L. C. . Enhanced Visible-Light Photocatalytic Activity of Anatase TiO_2_ through N and S Codoping. Appl. Phys. Lett. 98, 211903 (2011).

[b45] Wu J. M., Zhang T. W. & Zeng Y. W. Large-Scale Preparation of Ordered Titania Nanorods with Enhanced Photocatalytic Activity. Langmuir 21, 6995–7002 (2005). 16008414 10.1021/la0500272

[b46] Li H. X. . Mesoporous Au/TiO_2_ Nanocomposites with Enhanced Photocatalytic Activity. J. Am. Chem. Soc. 129, 4538–4539 (2007). 17381091 10.1021/ja069113u

[b47] Wu J. M. & Zhang T. W. Photodegradation of Rhodamine B in Water Assisted by Titania Films Prepared through A Novel Procedure. J. Photochem. Photobiol. A-Chem. 162, 171–177 (2004).

[b48] Yan X. L., Ohno T., Nishijima K., Abe R. & Ohtani B. Is Methylene Blue an Appropriate Substrate for a Photocatalytic Activity Test? A Study with Visible-Light Responsive Titania. Chem. Phys. Lett. 429, 606–610 (2006).

[b49] Zhang J., Xu Q., Feng Z., Li M. & Li C. Importance of the Relationship between Surface Phases and Photocatalytic Activity of TiO_2_. Angew. Chem. Int. Ed. 47, 1766–1769 (2008). 10.1002/anie.200704788 18213667

[b50] Xu Q. . Enhancing Hydrogen Production Activity and Suppressing CO Formation from Photocatalytic Biomass Reforming on Pt/TiO_2_ by Optimizing Anatase-Rutile Phase Structure. J. Catal. 278, 329–335 (2011).

[b51] Hurum D. C., Agrios G. A. & Gary A. Kimberly Explaining the Enhanced Photocatalytic Activity of Degussa P25 Mixed-Phase TiO_2_ Using EPR. J. Phys. Chem. B 107, 4545–4549 (2003).

[b52] Scanlon D. O. . Band Alignment of Rutile and Anatase TiO_2_. Nat. Mater. 12, 798–801 (2013). 23832124 10.1038/nmat3697

[b53] Li J. T. . Solar Hydrogen Generation by a CdS-Au-TiO_2_ Sandwich Nanorod Array Enhanced with Au Nanoparticle as Electron Relay and Plasmonic Photosensitizer. J. Am. Chem. Soc. 136, 8438–8449 (2014). 24836347 10.1021/ja503508g

[b54] Zheng Z. K. . Facile *in situ* Synthesis of Visible-Light Plasmonic Photocatalysts M@TiO_2_ (M = Au, Pt, Ag) and Evaluation of Their Photocatalytic Oxidation of Benzene to Phenol. J. Mater. Chem. 21, 9079–9087 (2011).

[b55] Jiang Z. F., Zhu C. Z., Wan W. M., Qian K. & Xie J. M. Constructing Graphite-Like Carbon Nitride Modified Hierarchical Yolk–Shell TiO_2_ Spheres for Water Pollution Treatment and Hydrogen Production. J. Mater. Chem. A 4, 1806–1818 (2016).

