# Supplementary material for: UV and visible light photocatalytic activity of Au/TiO2 nanoforests with Anatase/Rutile phase junctions and controlled Au locations
Source: Sci Rep. 2017 Jan 24;7:41253. doi: 10.1038/srep41253 (PMC5259751; doi:10.1038/srep41253)
Supplement: Supplementary Information [file srep41253-s1.pdf]

Electronic Supplementary Information for

UV and Visible Light Photocatalytic Activity of Au/TiO_2 Nanoforests with Anatase/Rutile Phase Junctions and Controlled Au Locations

Yang Yu, Wei Wen, Xin-Yue Qian, Jia-Bin Liu and Jin-Ming Wu*

State Key Laboratory of Silicon Materials and School of Materials Science and Engineering, Zhejiang University, Hangzhou 310027, P. R. China.

College of Mechanical and Electrical Engineering, Hainan University, Haikou, 570228, P. R. China.

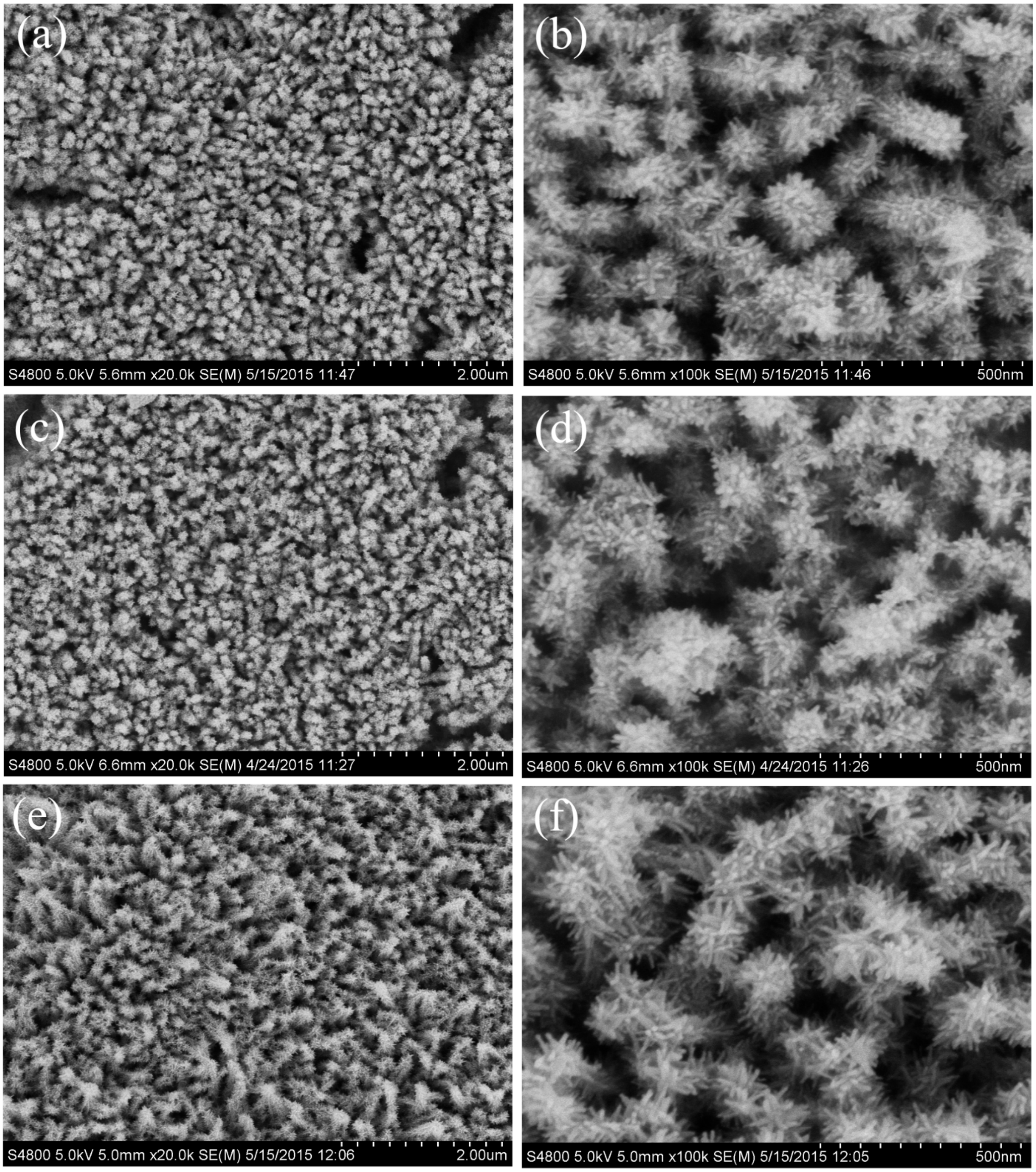

Figure S1. Low and high magnification top view FESEM images of the Branched-NW (a, b), Branched-NW-Au (c, d), and Branched-Au-NW (e, f).

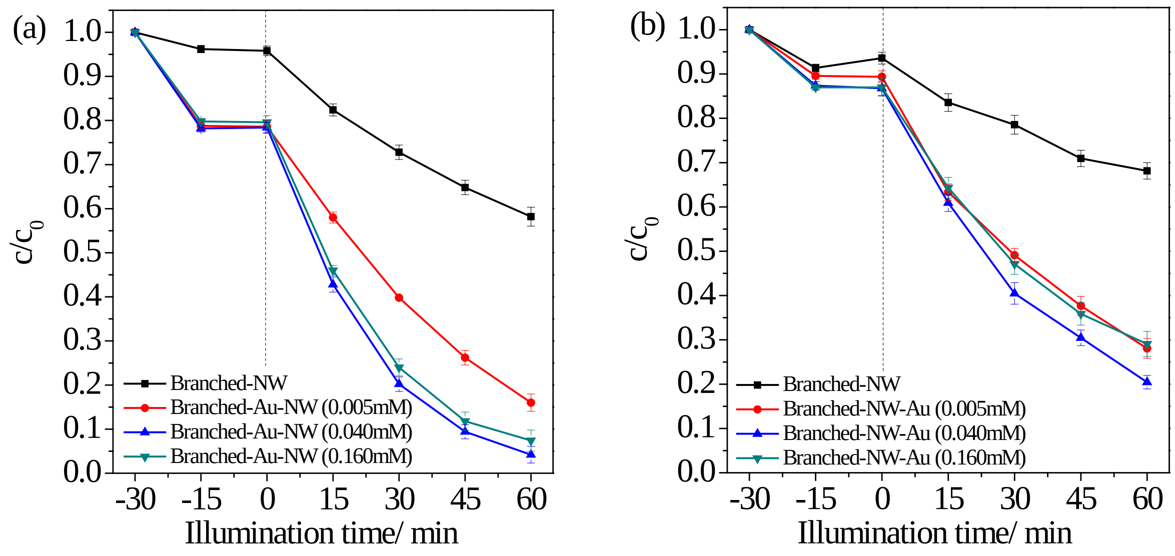

Figure S2. Photodegradation curves of rhodamine B in water under UV light illumination, in the presence of (a) the Branched-NW-Au and (b) Branched-Au-NW, for which the Au-loading was achieved using $HAuCl_4$ solutions with various concentrations.

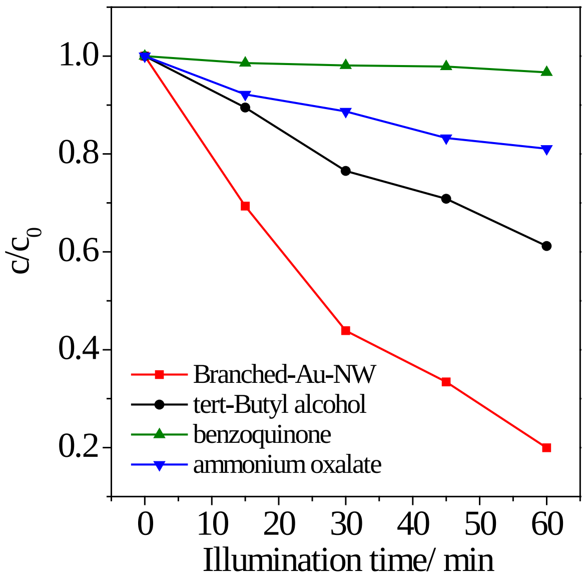

Figure S3. The effects of different quenching agents on the photodegradation procedures of rhodamine B in water in the presence of the Branched-Au-NW under the UV light illumination.

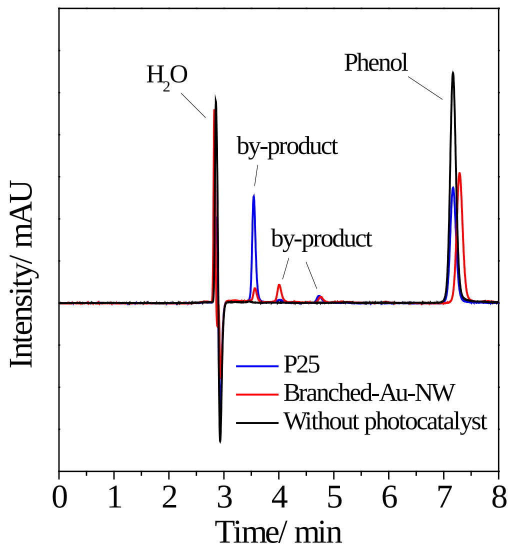

Figure S4. The LC spectra of phenol under the UV light illumination for 6 h, in absence and in the presence of the P25 film and the Branched-Au-NW.

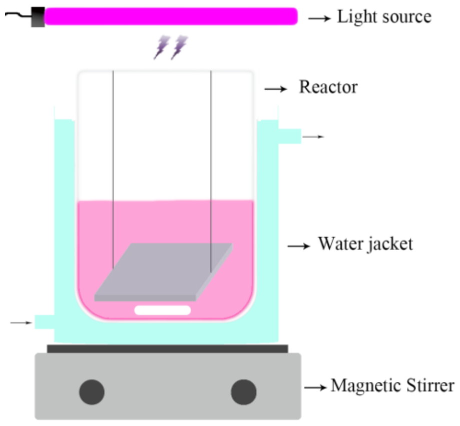

Figure S5. The schematic image showing the set-up of the photocatalytic tests.
